# Tc-99m HIG Scintigraphy in Detection of Active Inflammation in Ankylosing Spondylitis

**DOI:** 10.4274/MIRT.21

**Published:** 2011-08-01

**Authors:** Özhan Özdoğan, Berna Değirmenci, Özlem Şenocak, Selmin Gülbahar, Gülhan Arslan, Cengiz Taşçı, Elif Akalın, Hatice Durak

**Affiliations:** 1 Dokuz Eylül University, School of Medicine, Department of Nuclear Medicine, İzmir, Turkey; 2 Dokuz Eylul University, School of Medicine, Department of Physical, Therapy and Rehabilitation İzmir, Turkey

**Keywords:** Tc-99m-HIG, ankylosing spondylitis, inflammation

## Abstract

**Objective:** The diagnosis of active inflammation in ankylosing spondylitis (AS) is crucial for treatment to delay possible persistent deformities. There are no specific laboratory tests and imaging methods to clarify the active disease. We evaluated the value of Tc-99m human immunoglobulin (HIG) scintigraphy in detection of active inflammation.

**Material and Methods:** Twenty-nine patients were included. Tc-99m methylenediphosphonate bone (MDP) and HIG scintigraphies were performed within 2-5 day intervals. Two control groups were constituted both for MDP and HIG scintigraphies. Active inflammation was determined clinically and by serologic tests. Both scintigraphies were evaluated visually. Sacroiliac joint index values (SII) were calculated.

**Results:** Active inflammation was considered in five (sacroiliitis in 2, sacroiliitis-spinal inflammation in 1, achilles tendinitis in 1, arthritis of coxafemoral joints in 1) patients. HIG scintigraphy demonstrated active disease in all 3 patients with active sacroiliitis. But, it was negative in the rest. The other 2 active cases were HIG negative. Right and left SII obtained from HIG scintigraphy was higher (p<0.05) in clinically active patients than inactive patients. There was not any significant difference between patients with inactive sacroiliitis and normal controls. Right and left SII obtained from bone scintigraphy was higher (p<0.05) in patient group than in control group.

**Conclusion:** Clinically inactive AS patients, behave no differently than normal controls with quantitative sacroiliac joint evaluation on HIG scintigraphy. HIG scintigraphy may be valuable for evaluation of sacroiliac joints in patients with uncResults:ertain laboratory and clinical findings.

**Conflict of interest:**None declared.

## INTRODUCTION

Ankylosing Spondylitis is the prototype of spondyloarthropathies (1). It is a chronic systemic inflammatory disease that mainly affects the axial skeleton. The typical presentation of the disease is with low back pain of insidious onset. Arthritis of hips and shoulders and enthesopathies are common (2). The sacroiliac joints are always affected (3). The etiopathogenesis of AS is not clearly understood, although HLA-B27 is likely to play a central role (2). The skeletal pathology of AS is an inflammatory erosive process, evolving the enthesis, followed by new bone formation. 

Early and accurate diagnosis and effective patient education is essential for the treatment. Although the diagnosis is not difficult in the hands of an experienced clinician there is no specific test for the early diagnosis of AS and the reactivation of the disease (2). 

The laboratory tests are nonspecific for the diagnosis. Early in the disease the elevated erythrocyte sedimentation rate (ESR) reflects the inflammatory process but in chronic disease it may or may not be raised. No correlation between ESR and disease activity has been reported (2). Serum C-reactive protein (CRP) may be a better marker of disease activity in AS. Mild to moderate elevations of IgA and acute phase reactants (alpha-1-antitrypsin, alpha-1-acid glycoprotein and haptoglobulin) are frequently observed in AS (2). 

Typical radiological findings appear late in the course of the disease. Thus, use of conventional radiography in the diagnosis of the disease should be withheld (4). Computed tomography and gadolinium-enhanced magnetic resonance imaging are better for early diagnosis (5,6). MR may have some role on defining presence of active sacroiliitis (7). 

A multiphase bone scintigraphy helps to demonstrate increased vascularity and inflammation in AS. In the late disease, the inflammatory activity on the third phase of bone scintigraphy decreases (8,9,10). Bone scintigraphy demonstrates more lesions than conventional radiography in early disease. Although some quantitative approaches for bone scintigraphy were used, sacroiliac index is the most sensitive and gives characteristic values for AS (11), greater than 1.4-1.6 is indicative of sacroiliitis (12). Unfortunately, the specificity of quantitative bone scintigraphy is low because some diseases like hyperparathyroidism, lupus erythematosus and renal osteodystrophy may also show high SII values. 

^99m^Tc labeled nonspecific polyclonal human immunoglobulin (HIG) accumulates in infection and sterile inflammatory processes to such an extent that a target to background ratio develops permitting scintigraphic localization of an inflammatory process as in specific monoclonal immunoglobulins (13). HIG being a nonspecific marker of inflammation and infection may help to detect active inflammation in AS.

The aim of this study was to determine the value of HIG in demonstrating the disease activity in AS patients.

## MATERIALS AND METHODS

**Patients**

This study was approved by the local Ethical Committee. Written informed consent was obtained from all patients in accordance with Helsinki Declaration II.

Twenty-nine patients with the diagnosis of AS according to the New York criteria (2) were included in this prospective study. Patients with malignant musculoskeletal disorders and infectious or other inflammatory diseases were excluded from the study. 

We did not perform synovial biopsies for obtaining histopathological diagnosis of active inflammation due to ethical reasons. Instead some criteria were used by physical therapy and rehabilitation specialists to determine the disease activity. They were;

- On physical examination the signs of inflammation like tenderness, heat and edema were inspected in peripheric joints. 

- Specific clinical tests including “Mennel and Sacroiliac Compression” were performed for evaluation of sacroiliac joints and positivity of at least one is accepted to be indicative of disease activity. Schrober test was used to evaluate the vertebral column.

- In clinically suspicious cases, increased CRP values were accepted to be an activity criterion only in the absence of other pathologies that increase CRP value.

- Conventional radiographies and CT scans of the affected sites were obtained as indicated to help the clinical decision making and to rule out other pathologies. 

- The clinical decision was confirmed by evaluating the response to therapy with follow-up examinations for at least six months in all clinically active patients except one who has been lost during follow up.

**Imaging**

Tc-99m methylene diphosphonate (MDP) bone scintigraphy and HIG scintigraphy were performed in 2 to 5 days interval. 

A three-phase bone scintigraphy (AmerscanT Medronate II Agent) is performed in all patients except one. 925 MBq MDP was injected intravenously and blood flow, blood pool, whole body and static images (4^th^ hour) of the pelvis were obtained routinely. Additionally, blood pool and 4^th^ hour planar images of other body parts were obtained as indicated by symptoms and signs of the patients. 

Tc 99m HIG scintigraphy (DRN 4369 Technescan® HIG Mallincrodt Medical B.V. Petten, Holland) was performed following bone scintigraphy. The radiopharmaceutical (555 MBq) was injected intravenously. Anterior and posterior planar images of the pelvis and other body parts (as indicated by symptoms and signs of the patients) were obtained during the blood pool phase (10 min.), 4th hour (10 min.) and 24th hour (15 min). Whole body images were obtained during blood pool phase and at 4^th^ hour. A Siemens Multispect II gamma camera with a 20% window set at a 140 keV energy peak was used for imaging. A parallel hole, low energy, general-purpose collimator was used for dynamic studies and the images were recorded as a 64X64 pixel matrix. A parallel hole, low energy, high resolution collimator was used for planar and whole body imaging and the images were recorded as a 256X256 pixel matrix.

For quantitative analysis two age matched control groups were constituted for MDP (control 1) and HIG (control 2) scintigraphies. Patients who were referred to bone and HIG scintigraphies without any known rheumatic diseases and pelvic pathologies constituted the control group. There were 22 (14 men, 8 women; age range, 30-71 y; mean age 50.9±10.8) and 12 (6 men, 6 women; age range, 38-65 y; mean age 46.9±7.2) patients in groups control 1 and control 2 respectively. 

**Image Analysis**

**Figure 1:**
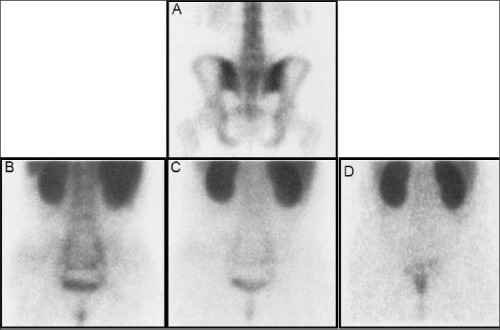
Posterior static images of a control patient withphysiological HIG distribution (A: 4^th^ hour bone scan, B: blood poolHIG, C: 4^th^ hour HIG and D: 24^th ^hour HIG )

The images were analyzed visually and quantitatively. Two nuclear medicine physicians who were blinded to the clinical scores and laboratory findings evaluated both scintigraphies visually. The sacroiliac joints of the patients in the control group were evaluated first. The disease activity in patient group was determined by comparing the patient images with the images of control group. Increased HIG accumulation on early images persisting on late images (24^th^ hour images) was accepted as active inflammation visually. The posterior pelvic images of a control group patient are given in Figure 1. The HIG accumulation on sacroiliac joints was within normal range. 

**Figure 2:**
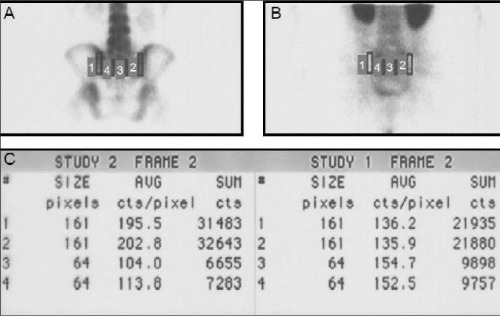
Posterior static images of a control patient withphysiological HIG distribution (A: 4^th^ hour bone scan, B: blood poolHIG, C: 4^th^ hour HIG and D: 24[ref:th]th[/ref] hour HIG )

For quantitative evaluation region of interests were drawn on sacroiliac joints and sacrum of the patients and controls on both 4^th^ hour MDP and HIG scintigraphies (Figure 2) as defined by Goldberg et al (14). Sacroiliac to sacral count ratios (sacroiliac indices – SII) were obtained which was defined to be an effective quantitative method for AS patients by Schorner and et al (15).

The differences in measured parameters between the patients and control groups, visual and quantitative parameters of MDP and HIG were analyzed with Mann–Whitney U test. The correlations of patient group SII with patients’ exercise program, medical treatment (either first line, second line or combined), and CRP, ESR values were tested using Spearman correlation analysis. A p value <0.05 was considered as statistical significance.

## RESULTS

There were 29 patients in the study group (20 men, 9 women; age range, 30-71 yr; mean age 46.1±10.2). AS was diagnosed 1 week to 41 years ago (mean duration of illness: 10.8±9.1). Nine of the patients were obeying their exercise program regularly (31%). On the other hand the rest of the patients (69%) either were not performing an exercise program (17 patients) or were on irregular exercise (3 patients). Seven of the patients (24%) were not taking any medications while 9 patients were using first line, 6 patients were using second line medications and the remaining 7 patients were using both. 

ESR and CRP were both high in 17 patients (59%). CRP alone was high in 6 and ESR in 2. Seventeen of the patients had HLA-B27 analysis and thirteen were HLA-B27 positive (76%). 

Active disease was detected in 6 different anatomic locations (joints) in 5 patients (17%) by clinical assessment. The six anatomic locations of clinically active disease were the sacroiliac joints in 3 patients, vertebral column, bilaterally the coxa-femoral joints and finally the right heel (Achilles tendinitis) in one patient (Table 1). 

**Figure 3:**
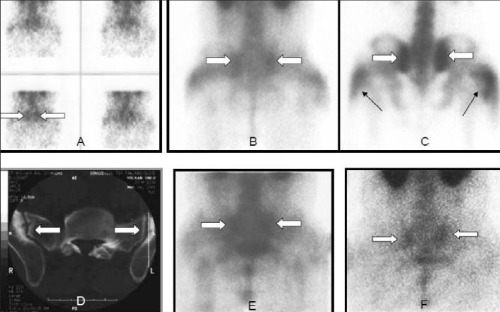
A 32-y-old male with clinically active sacroiliitis. Flow (A),blood pool (B), 4^th^ hour planar (C) images of bone scan. CT (D), 4thhour (E) and 24^th^ hour (F) HIG images of pelvis. Note the increasedflow, hyperemia and osteoblastic activity in sacroiliac joints on bonescintigraphy (white arrows). Increased HIG accumulation andretention were concordant with the clinical findings in this patient.Degenerative changes of both sacroiliac joints accompanied withsubchondral sclerosis and small calcifications were reported on CT(white arrows). The bilateral gluteal uptake is due to the repeatedintramuscular injections (black arrows)

**Figure 4:**
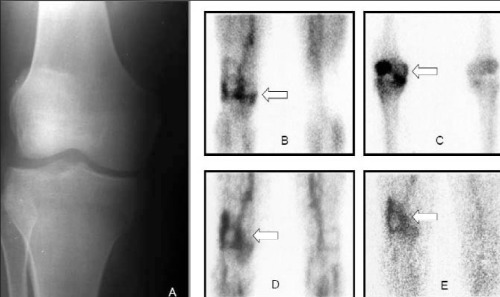
A 32-y-old male with the diagnosis of AS for 4 years. Thepatient was complaining of pain on the right knee. ESR and CRPwere found to be high but there was not active inflammation inclinical evaluation. Radiography (A), blood pool (B) and 4^th^ hour planarMDP (C) images, 4^th^ hour (D), 24^th^ hour HIG (E) images (bothanterior views). A marked hyperemia over the right knee joint andosteoblastic activity over patella, medial epicondyl, proximal tibia wasobserved on bone scan. HIG scan was similar to bone scan exceptthat the hyperemia and HIG accumulation on 4th hour images weremore prominent in lateral side of distal femur indicating a possibleinflammation of the tendon (arrows)

On visual assessment HIG scintigraphy was positive bilaterally in all clinically active sacroiliac joints (Figure 3). HIG was negative in the vertebral column of the patient with active sacroiliitis (patient number 2, Table 1) and was also negative in the remaining 2 patients with clinically active disease of Achilles tendon and coxa-femoral joint. On visual assessment HIG was false positive in 3 patients. These false positive results were observed in thoracic vertebra (2 patients) and in sacroiliac joints and in the right knee joint of a patient (Figure 4). The results of HIG scan were also given in Table 1.

On visual assessment hyperemia was observed in the sacroiliac joints bilaterally in all patients with clinically active sacroiliitis on bone scans. It was negative in the Achilles tendon and in the coxa-femoral joint of clinically active patients. Additionally hyperemia was also observed in some levels at the thoracic spine of a patient with clinically active sacroiliitis but without any active disease in the spine. The findings on blood pool phase of the bone scan were very similar with the HIG results. The late phase of bone scans was positive in the sacroiliac joints of the 2 patients with clinically active sacroiliitis and negative in the one. Late phase bone scan was also positive in the coxa-femoral joint of the patient with active disease but negative in the Achilles tendon of the other patient (Table 1). 

The mean SII values on the left and right sacroiliac joints in bone scintigraphy were 1.47±0.20 (min: 1.09, max: 1.79) and 1.48±0.22 (min: 1.13, max: 1.90) in patient group and 1.32±0.18 (min: 1.00, max: 1.64), 1.29±0.17 (min: 0.95, max: 1.68) in control group, respectively. The mean SII was higher in the patient group (Table 2) than in the control group (right SII: p<0.005 and left SII: p<0.014).

The mean SII values of HIG scintigraphy were 1.05±0.14 (min: 0.79, max: 1.27) and 0.96±0.12 (min: 0.77, max: 1.24) in the patient group and 1.03±0.10 (min: 0.92, max: 1.27), 0.93±0.15 (min: 0.79, max: 1.25) in control group on the left and right sacroiliac joints, respectively (Table 2). There was no statistical difference between the patient and control group. On the other hand there was a statistically significant difference between the left and right SII values in each group (p<0.05). Left SII values are greater than right both in patients with active sacroiliitis, inactive patients and control group (Table 3).

There was not any correlation between bone and HIG scintigraphy SII values in the patient group. We did not find any correlation between HIG scintigraphy SII values and patients’ exercise program, medical treatment (NSAID and/or SLZ), ESR, CRP values. 

The major limitation of this study was the small number of the patients with clinically active disease. That’s why we abstained from calculating sensitivity and specificity values of HIG scintigraphy in detection of active disease.

## DISCUSSION

According to general acceptance immunologic mechanisms play a major role in the pathogenesis of peripheric inflammatory arthropathy and sacroiliitis observed in AS patients (3). HIG was shown to be a reliable method to localize inflammation in rheumatoid arthritis (16) and some other infectious and noninfectious inflammation (17,18,19,20,21). So, we investigated the role of HIG in detecting active disease in AS patients. In our study increased activities on sacroiliac joints suggesting an active inflammation were detected in all patients with clinically active sacroiliitis. Contrary to our findings, Leslie W.D and his co-workers have demonstrated that there was no difference in HIG uptake by visual and quantitative analysis among patients with clinical active sacroiliitis and control rheumatologic patients, claiming that the close proximity of iliac vessels, gut and urinary bladder activities to the sacroiliac joints makes quantitative evaluation difficult (22). The number of patients with active sacroiliac disease is limited in both studies. This may be the reason for the incompatibility of the two studies. In our limited number of patients we observed increased HIG accumulation visually in the clinically active patients. Quantitative analyses of sacroiliac joints are difficult on HIG scintigraphy because the bone visualization is poor and it is difficult to discriminate sacrum and the sacroiliac joints in order to draw region of interests. That’s why we used the posterior pelvis images of the bone scintigraphy as a reference for drawing ROIs to HIG scintigraphies. Using this method the SII in patients with active disease was also higher then the SII of the patients with inactive sacroiliitis on quantitative analyses. 

All patients who had clinically active sacroiliac disease also demonstrated hyperemia in blood pool images on bone scan. The agreement of HIG and blood pool phase of bone scintigraphy was also previously demonstrated in the diagnosis of synovitis (23). Both blood pool phases of bone and HIG scintigraphies were able to diagnose active sacroiliitis in our limited number of patients. This finding may be attributed to the most widespread accepted mechanism, which is the leakage of HIG to expanded extracellular space through vascular endothelium with increased permeability (24). Nevertheless, the mechanism of IgG localization in inflammatory area is not exactly known. Binding of IgG to Fc receptors on inflammatory cells and the specific monocyte associated uptake mechanism were some proposed mechanisms. But, quantitative microautoradiographic studies showed that HIG preferentially localizes at edematous interstitial spaces of infected tissue rather than inflammatory cells and In-111 HIG was demonstrated to accumulate in neutropenic rats (25). 

The late phase of bone scintigraphy cannot differentiate the inflammatory activity from a degenerative disease. That’s why we observed more lesions in late phase of bone scan than the blood pool images and HIG scan. Other scintigraphic methods like Tc-99m HMPAO leukocyte scintigraphy which was used to detect inflammatory bowel disease associated with spondyloartropathies (26) are not indicated in inflammatory joint diseases. Tc-99m pyrophosphate and Tc-99m nanocolloid used in detecting inflammatory processes in AS were shown to be neither sensitive nor specific to diagnose sacroiliitis (27). 

There was a statistically significant difference of SII values on bone scintigraphies between patient and control group. We believe that sclerotic and calcific degenerative changes secondary to the sacroiliitis was the cause for the difference. The presence of a major overlap between patients and controls (Table 2) was also an important finding, which makes it impossible to separate normals from abnormals by quantitative means with bone scintigraphy. The same overlapping is also true for the SII obtained from HIG scintigraphies (Table 2). 

The SII values obtained on HIG scintigraphy was very similar in clinically inactive AS patients and the controls. Clinically inactive patients behave no differently from normal controls on HIG scintigraphy (Table 3). But, the SII values of patients with clinically active sacroiliitis were significantly higher than the inactive patients and the control group (Table 3). The HIG scan in this limited number of patients was able to detect the inflammatory response of active inflammation. 

We encountered some unexpected results. The SII values on the left side on HIG scintigraphy were statistically higher than the right side both in patients and the control group (Table 3). But, there was not any difference of SII values between left and right sides on bone scintigraphy. We don’t believe that the difference is due to a systematic error of ROIs. We draw ROIs to sacroiliac joints in HIG scintigraphy by using bone scintigraphy as the reference. We believe that the difference can be attributed to the asymmetrical shine-through of iliac vessels causing superimposition of blood, due to prolonged blood pool to the left sacroiliac joint. The presence of this asymmetrical shine-through seems to be a drawback for the use of HIG for both visual and quantitative analysis of sacroiliac joints. Using SPECT images for sacroiliac joint evaluation may overcome this problem. 

Both blood pool phase of bone scan and HIG scintigraphies were negative at Achilles tendon and coxa-femoral joints of patients with clinically active disease. We also could not demonstrate HIG accumulation in the patient who had active spinal inflammation clinically. K. de Vlam and his co-workers had also studied MDP and HIG scintigraphies in some AS and osteoarthritis patients. They reported that HIG SPECT was not useful for determination of inflammation due to marrow uptake and not useful in spinal inflammation in AS patients (28). We also experienced high marrow uptake in some patients. High marrow uptake, regardless of the cause, has the potential of creating "quantitative false positives". We believe that HIG has limited value in demonstrating spinal inflammation because the anterior border of vertebral body which is the most frequent site of spinal inflammation in AS patients has poor blood supply. Additionally the vertebral joints are small and the presence of the high physiologic uptake of neighboring tissues like the liver, the kidneys and the spleen makes the evaluation difficult. 

The limited spatial resolution of gamma cameras and the postural disturbances in AS patients, the low specificity of HIG scan, and the presence of the neighboring organs with high blood pool activity were the limitations of HIG scintigraphy in the detection of existing activities in AS patients. 

HIG positive, clinically negative patients (false positive cases) were followed up at least six months and never diagnosed to have clinically active disease although they experienced some pain in HIG positive sites during the course of follow up. 

## CONCLUSION

HIG scan demonstrated increased uptake on sacroiliac joints of all 3 patients with clinically active sacroiliitis. Although the number of patients with clinically active sacroiliitis is very limited, there is enough evidence to state that the clinically inactive AS patients, behave no differently than normal controls with quantitative sacroiliac joint evaluation on HIG scintigraphy. Therefore HIG scintigraphy may help to differentiate active inflammation of sacroiliac joints in patients with uncertain laboratory and clinical findings. Further studies including a larger patient group with active disease are needed.

**Acknowledgments **

The author wishes to thank Özden Ülker and Türkan Ertay for their help in preparing radiopharmaceuticals and to the technicians of the department. 

## Figures and Tables

**Table 1:**
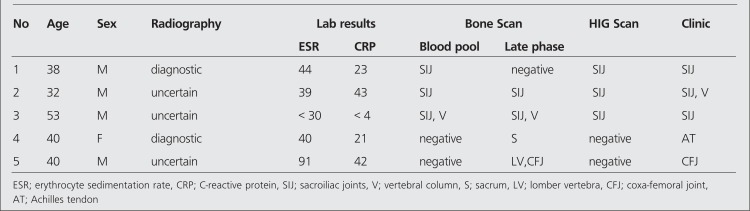
The demographic, radiographic and scintigraphic data of five patients who have clinically active disease

**Table 2:**
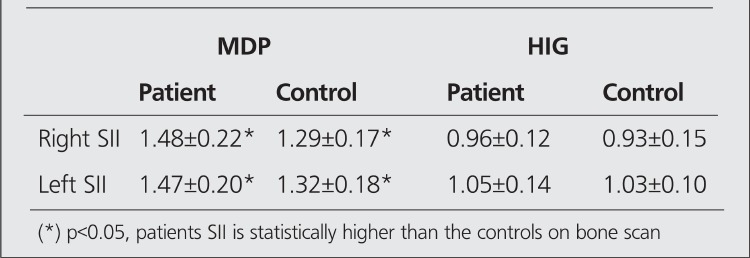
Quantitative analysis, bone and HIG scans

**Table 3:**
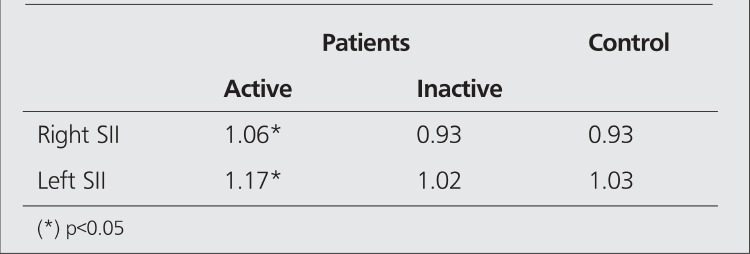
Quantitative analysis of SII values on HIG scan
